# The Regulatory Challenges for Drug Repurposing During the Covid-19 Pandemic: The Italian Experience

**DOI:** 10.3389/fphar.2020.588132

**Published:** 2020-09-25

**Authors:** Lucia Gozzo, Laura Longo, Daniela Cristina Vitale, Filippo Drago

**Affiliations:** ^1^Clinical Pharmacology Unit, Regional Pharmacovigilance Centre, University Hospital of Catania, Catania, Italy; ^2^Department of Biomedical and Biotechnological Sciences, University of Catania, Catania, Italy

**Keywords:** COVID19, off-label, drug repurposing, emergency law, Italian regulatory system

## Introduction

The search for safe and effective treatments for Covid-19 started early and focused in particular on drug repurposing of available molecules in the hope of finding valuable therapeutic options as quickly as possible.

As reported on *Covid19db*, a free online database of trials of medicinal products to prevent or treat Covid-19, the percentage of trials including repurposed drugs exceeds 60% of the total number of interventional drug-based trials (AnticancerFund; Pan Pantziarka et al., 2020).

The main advantages of drug repurposing over *de novo* medicine research are the faster and potentially cheaper development and the reduced risk of failure due to safety concerns (Bertolini et al., 2015; Pushpakom et al., 2019; Verbaanderd et al., 2019). Therefore, the regulatory authorities were rapidly overwhelmed by requests for clinical trials and compassionate use program approval.

One of the national regulatory agencies that has suffered the most because of the Covid-19 crisis has been the Italian one.

Moreover, in a situation of absolute emergency with hospitals full of critical patients, clinicians tried to save lives with off-label drugs used according to the available (although weak) evidence.

## Clinical Trials Approval: Successful Attempt of Centralization

The Italian Medicines Agency (AIFA) is the Competent Authority for issuing the authorization of all clinical trials of medicinal products together with the local Ethics Committees (ECs) competent for the clinical sites for their formal approval (**Supplementary Appendix 1A**). As reported in a national registry (AIFA, 2019b), despite the progressive reduction established by a Decree (Decree-Law, 2013) 90 ECs are still working in Italy, which should be further reduced to 40 local ECs and three national ones (Petrini and Brusaferro, 2019) in order to optimize their performance and improve efficiency. This condition can indeed produce disparities in terms of opinions and/or timing for approval.

On an exceptional basis, during the Covid-19 crisis an emergency regulation for clinical trials and compassionate uses was issued with the Law Decree N. 18 on 17 March 2020 (Decree-Law, 2020). According to the aforementioned Decree, Covid-19 protocols have to be preliminarily evaluated and subsequently approved by the Technical Scientific Committee (CTS) of AIFA, by the AIFA Clinical Trial Office and by the EC of the National Institute for Infectious Diseases Lazzaro Spallanzani in Rome, as single ECs, which express a nationwide opinion (**Supplementary Appendix 1B**). The rationale of the measure was to speed up the approval process thanks to a single national body (instead of the multitude of ECs usually involved based on territorial criteria) and to guarantee a high level of quality of the assessment thanks to the expertise of the institution on the treatment of infectious diseases. Moreover, the Decree established that both AIFA and Istituto Spallanzani are responsible for the activation of Covid-19 compassionate use programs (CUPs), which must be assessed and eventually authorized as a matter of urgency.

One month from these provisions, AIFA published a summary report on the work performed by the Commission and the national EC (AIFA, 2020d). In the reference period, 80 Covid-19 protocols and proposals had been assessed, and 16 positive opinions (20%) had been issued. The main reasons behind the rejections were concerns about the study design, the rationale, and a not adequately defined population.

If we look at the clinical trials approved by AIFA under normal conditions, in the last published report (AIFA, 2019a) we find 714 protocols assessed during 2018 (almost 60 per month), of which 666 had been approved (93.3%). Probably the highest percentage of positive opinion is related to the highest quality of submission under normal circumstances.

According to the latest update, about 40 clinical trials for Covid-19 have been approved in Italy out of a total of 156 protocols submitted (Popoli, 2020) (**Supplementary Appendix 2**), even in light of the difficulty of completion due to the reduction in the number of new cases and hospitalizations.

Despite the expected complexity of studies management due to the emergency, well-designed clinical trials were favored. Indeed, the great majority of approved studies were randomized–controlled trials (**Supplementary Appendix 3**), even if less than 25% were blinded. However, a critical point was undoubtedly the definition of the *“standard of care”* in the absence of authorized treatments. In order to guide clinical practice and trial design, AIFA published and periodically updated reports concerning drugs recommended according to national and international guidelines (AIFA, 2020a).

Other measures have been put in place to ensure the safe conduct of clinical trials and promote research on Covid-19.

Due to the exceptional restrictive measures introduced in order to fight the Covid-19 pandemic and in line with European directions (EMA, 2020b), AIFA provides indications regarding the management of all clinical trials that must be conducted with the highest protection of participants and maintaining adequate supervision even during emergencies (AIFA, 2020b). For instance, sponsors were invited to implement a risk-proportionate action plan, in view of the need to minimize contact between patients and the investigational team, and not to overload healthcare facilities. Moreover, carrying out procedures directly at the patient’s home may be considered. On-site monitoring visits can be replaced by exceptional and alternative monitoring such as telephone calls or video-calls with the trial site staff, or can be postponed.

Finally, the Italian Ministry of Health recently authorized the financial support of 10 important research projects on Covid-19 (Ministry of Health, 2020).

## Off-Label Use for Covid-19 and Emergency Approval

According to the European Medicines Agency (EMA), off-label use “relates to situations where the medicinal product is intentionally used for a medical purpose not in accordance with the authorized product information” (EU, 2017).

A major advantage of off-label use is the potential and rapid satisfaction of medical needs, especially in cases where no other options are available, even if it could increase the risk of inappropriate use and medical error due to the lack of a defined risk-benefit ratio (Bellis et al., 2014). Therefore, appropriateness in off-label drug prescriptions must be carefully assessed in order to ensure this use occurs only in the presence of data supporting a favorable risk/benefit profile.

Off-label prescribing is not currently regulated by European Union (EU) legislation, but some countries have adopted specific laws (**Supplementary Appendix 4**) (EU, 2017).

Italy has gained a lot of experience in off-label regulation and management as a result of the so-called “*Di Bella case*” (Di Bella, 2010). In order to limit off-label use, to guarantee patients’ well-being and reduce unmotivated risks, the Italian Parliament issued Law 94 in 1998, which allows physicians to perform off-label prescriptions in individual and exceptional cases, provided that the following requirements are respected:

- the assumption of responsibility of the prescriber,
- an adequate informed consent of the patient,
- and the existence of scientific evidence of the efficacy and safety of the medicine derived from at least phase II clinical trials (Financial-Law, 2008).

Moreover, the Law establishes that the National Health System (NHS) does not cover the cost of treatment, which must be granted by patients themselves. In a hospital setting, prescribers must request the authorization for off-label treatment to the local Health Director, and costs are covered by the hospital budget.

The only case in which the Italian NHS can reimburse an off-label drug is its use under Law 648/1996 as reported in specific lists, updated based on new scientific evidence resulting from at least phase II clinical trials (Law 648, 1996).

**Figure 1:**
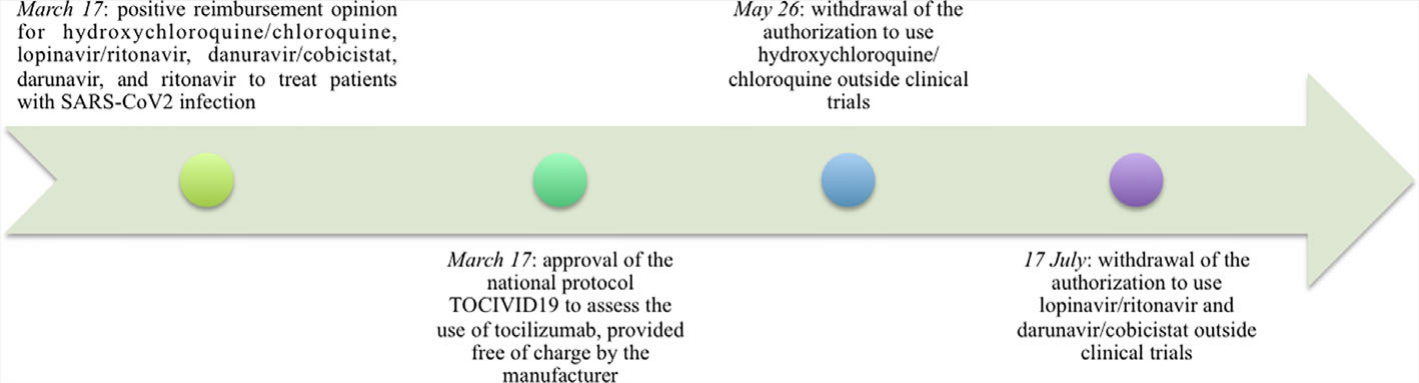
Timeline of the main AIFA opinions on off-label drug use issued during the Covid-19 emergency.

The Covid-19 emergency obliged national authorities to consider the possibility to allow a systematic off-label use of some medicines notwithstanding the aforementioned rules (**Figure 1**). In particular, this happened for hydroxychloroquine/cloroquine, lopinavir/ritonavir, and darunavir/cobicistat, whose use in patients with Covid-19 was promptly and provisionally approved for reimbursement, despite the non-applicability of the Law 648/1996 (above all due to the lack of evidence from phase II clinical trials), in order to manage their uncontrolled off-label use, which was already spreading nationwide. This decision allowed the standardization of prescriptions giving official instructions on how to use these medicines, but also to carry out an appropriate surveillance because of the obligation for prescribers to promptly share data about treated patients.

Recently, AIFA published the Report on Medicines used during the Covid‐19 epidemic showing a very high increase of hydroxychloroquine use compared to January 2019, a sign of clinicians hopes for this drug in the absence of available alternatives (AIFA, 2020e).

Subsequently, due to the lack of evidence and the possible risk of serious adverse events (Boulware et al., 2020; Cao et al., 2020; EMA, 2020a; FDA, 2020; Lother et al., 2020; Mehra et al., 2020a; Mehra et al., 2020b; WHO, 2020a; WHO, 2020b), AIFA revoked the authorization.

It is noteworthy that the next drug in terms of use following the anti-malarial is the antibiotic azithromycin, the use of which has never been officially authorized outside clinical trials (AIFA, 2020e; AIFA, 2020a). These findings deserve further analyses.

The case of tocilizumab is different, it has been provided free of charge by the Company since the beginning of March. In this case, in order to monitor all patients treated with the drug and to collect solid real-world data, AIFA together with Istituto Nazionale Tumori, IRCCS, Fondazione G. Pascale (Napoli) promoted a nationwide trial that involved hundreds of clinical centers and enrolled thousands of patients (AIFA, 2020c). The final results of the study are expected to be published in the near future.

## Conclusion

The Covid-19 pandemic tested the regulatory authorities’ ability to react to an emergency. In this context, Italy promptly implemented many measures (including centralization of clinical trials approval, simplification of the trial management obligation, financial support for research proposals, off-label use funding and governance) in order to simplify the practice of drug repurposing but also to maintain a strict control on drug access. Although some decisions were later withdrawn, the Italian regulatory authority was vigilant, efficient, and adaptable to face such a great challenge. Moreover, centralization has proven to be a successful choice, and a way forward in the future, albeit perfectible.

This success can be useful in order to start reviewing some old regulations and to further simplify some procedures, to make the system competitive and guarantee equal access to patients.

Finally, a dialogue among European member states and other authorities worldwide is desirable to set common criteria for proper off-label use management.

## Author Contributions

LG wrote the first draft of the manuscript. FD checked and revised the draft manuscript. All authors contributed to the article and approved the submitted version.

## Conflict of Interest

The authors declare that the research was conducted in the absence of any commercial or financial relationships that could be construed as a potential conflict of interest.
