# Supplementary material for: The Regulatory Challenges for Drug Repurposing During the Covid-19 Pandemic: The Italian Experience
Source: Front Pharmacol. 2020 Sep 25;11:588132. doi: 10.3389/fphar.2020.588132 (PMC7546760; doi:10.3389/fphar.2020.588132)
Supplement: Supplementary file 1 [file DataSheet_1.pdf]

Appendix 1a: workflow for clinical trial authorization in Italy and number of local ethics committes per region. *EC = Ethic Committee; AIFA = Italian Medicines Agency*

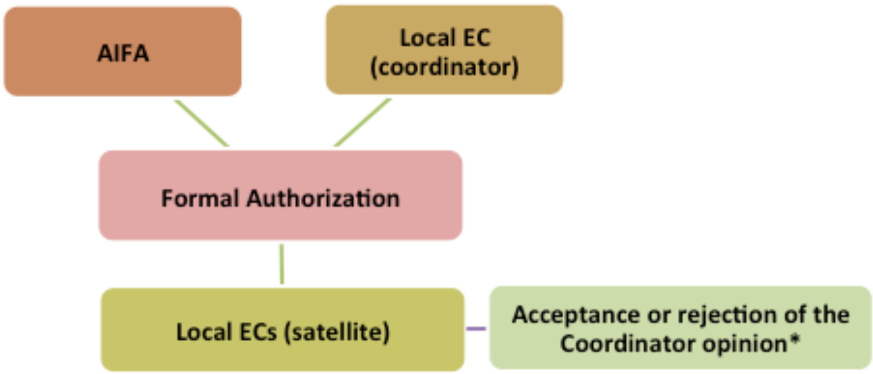

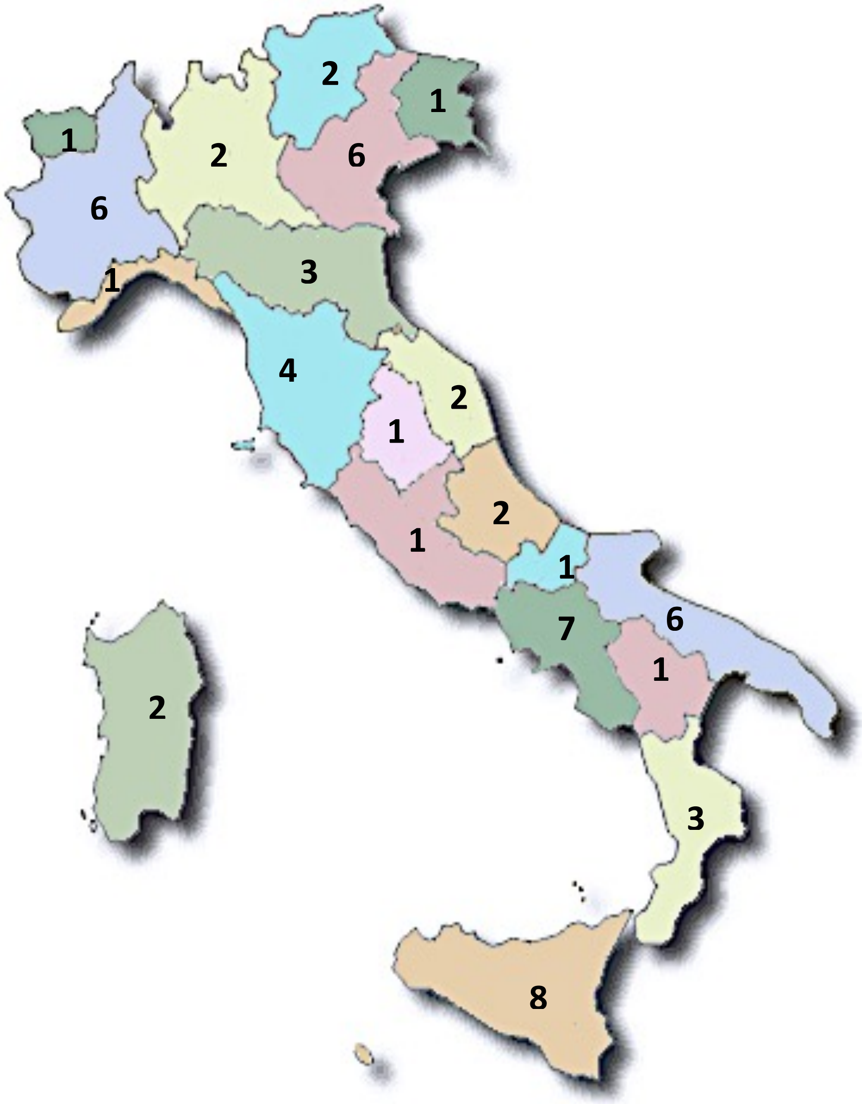

*The satellite ECs have the possibility to request changes/additions exclusively to the center-specific documentation (insurance policy, economic agreement, informed consent, limited to subjects undergoing trials at their center). This process can produce local disparities in terms of opinions and/or timing for approval.

Appendix 1b: workflow for COVID-19 clinical trial authorization in Italy (Law Decree N. 18/2020). *EC = Ethic Committee; AIFA = Italian Medicines Agency;* ***CTS*** *= Technical Scientific Committee*

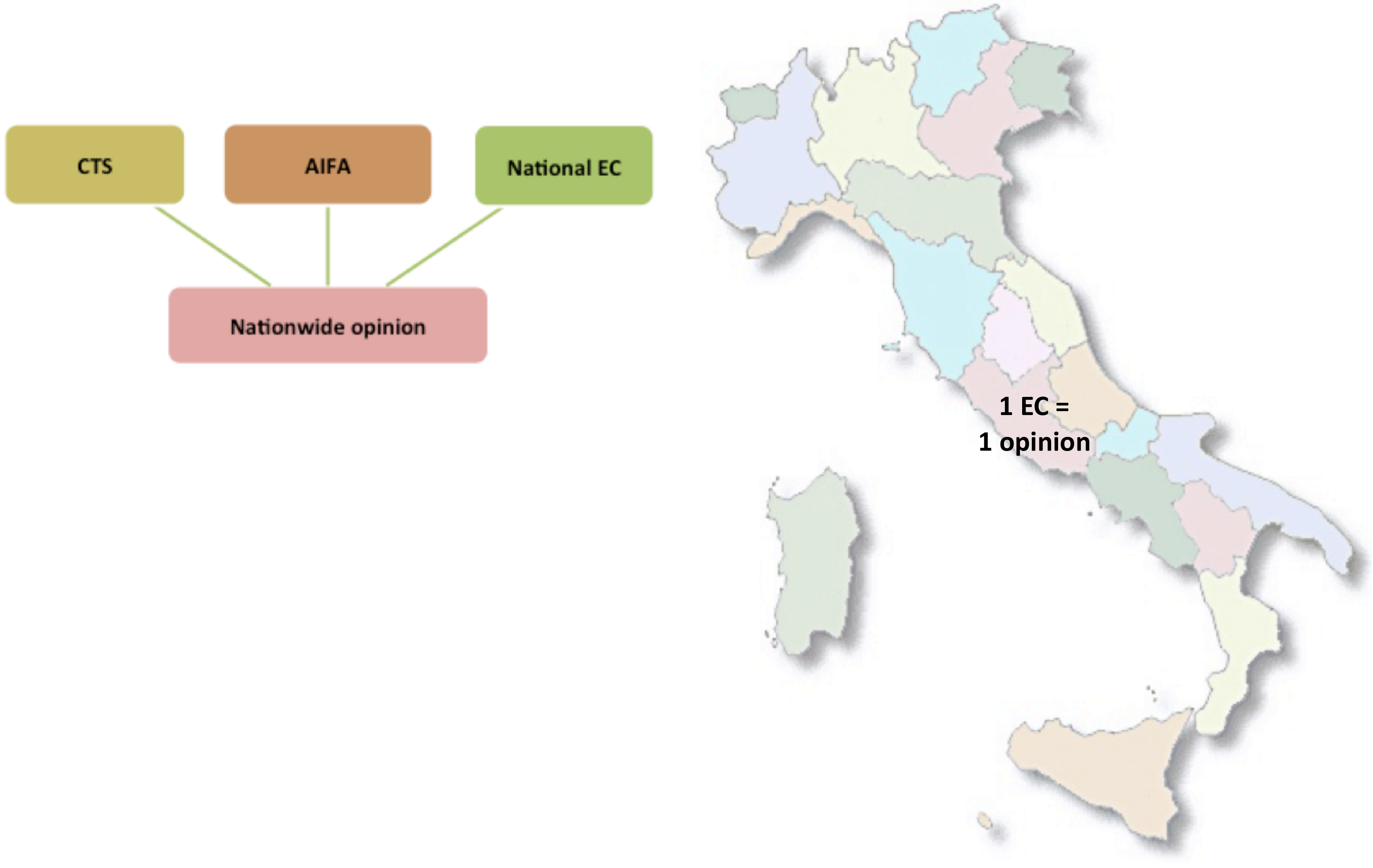

Appendix 2: clinical trials approved in Italy for COVID-19 (adapted from https://www.aifa.gov.it/en/web/guest/home)

	Drug	Title	Phase	Promotor
1	ABX464	A phase 2/3, randomized, double blind, placebo-controlled study to evaluate the efficacy and the safety of ABX464 in treating inflammation and preventing COVID-19 associated acute respiratory failure in patients aged ≥ 65 and patients aged ≥18 with at least one additional risk factor who are infected with SARS-CoV-2. (the MiR-AGE study).	II/III	ABIVAX
2	Acalabrutinib	A Phase 2, Open Label, Randomized Study of the Efficacy and Safety of Acalabrutinib with Best Supportive Care Versus Best Supportive Care in Subjects Hospitalized with COVID-19	II	Acerta Pharma BV
3	Baricitinib	BARICIVID-19 STUDY: MultiCentre, randomised, Phase IIa clinical trial evaluating efficacy and tolerability of Baricitinib as add-on treatment of in-patients with COVID-19 compared to standard therapy	II	Azienda Ospedaliera Universitaria Pisana
4	Baricitinib	A proof-of concept study of the use of Janus Kinase 1 and 2 Inhibitor, Baricitinib, in the treatment of COVID-19-related pneumonia	II	Fondazione IRCCS Policlinico S. Matteo, Pavia
5	Canakinumab	Phase 3 multicenter, randomized, double-blind, placebocontrolled study to assess the efficacy and safety of canakinumab on cytokine release syndrome in patients with COVID-19-induced pneumonia (CAN-COVID)	III	Novartis Research and Development
6	Colchicine	Treatment with COLchicine of patients affected by COVID-19: a pilot study (COLVID-19)	II	Azienda Ospedaliera di Perugia
7	Colchicine	Colchicine to counteract inflammatory response in COVID-19 pneumonia	II	Azienda Ospedaliero-Universitaria di Parma
8	Colchicine	ColcHicine in patients with COVID-19: a home CarE study	III	Società Italiana di Reumatologia
9	Chloroquine/ hydroxychloroquine	Chloroquine/ hydroxychloroquine prevention of coronavirus disease (COVID-19) in the healthcare setting; a randomised, placebo-controlled prophylaxis study (COPCOV)	/	University of Oxford

10	Darunavir-cobicistat<br>Hydroxychloroquine<br>Lopinavir-ritonavir<br>Favipiravir	Adaptive Randomized trial for therapy of COrona virus disease 2019 at home with oral antivirals (ARCO-Home study)	III	INMI "L. Spallanzani" - Roma
11	Defibrotide	Use of Defibrotide to reduce progression of acute respiratory failure rate in patients with COVID-19 pneumonia	II	IRCCS Ospedale San Raffaele – Milano
12	Emapalumab<br>Anakinra	A phase 2/3, randomized, open-label, parallel group, 3-arm, multicenter study investigating the efficacy and safety of intravenous administrations of emapalumab, an anti-interferon gamma (anti-IFNγ) monoclonal antibody, and anakinra, an interleukin-1(IL-1) receptor antagonist, versus standard of care, in reducing hyper-inflammation and respiratory distress in patients with SARSCoV-2 infection (Sobi.IMMUNO-101)	II/III	Swedish Orphan Biovitrum AB, Stockholm, Sweden
13	Enoxaparin	Intermediate dose enoxaparin in hospitalized patients with moderate-severe COVID19: a pilot phase II single-arm study, INHIXACOVID19	II	Università di Bologna
14	Enoxaparin	Prophylactic or therapeutic doses of enoxaparine and monitoring of outcomes in Covid-19 infected subjects: pilot study on 300 cases enrolled by a single institution - EMOS-COVID study	III	ASST-FBF-SACCO
15	Enoxaparin	Enoxaparin for thromboprophylaxis in hospitalized COVID-19 patients: comparison of 40 mg od versus 40 mg bid. A randomized clinical trial. X-COVID	III	ASST Grande Ospedale Metropolitano Niguarda
16	Favipiravir	A Multi-center, Randomized, Double-blind, Placebo-controlled, Phase III Clinical Study Evaluating the Efficacy and Safety of Favipiravir in the Treatment of Adult Inpatients with COVID-19-General Type (HS216C17	III	ASST Fatebenefratelli Sacco
17	Hydroxychloroquine	Hydroxychloroquine sulfate early administration in symptomatic out of hospital COVID-19 positive patients (Hydro-Stop-COVID19 Trial)	II	ASUR-AV5 Ascoli Piceno
18	Hydroxychloroquine	PRECOV: single-blind controlled study on the effects of hydroxychloroquine in the prevention of COVID-19 in health care workers at risk	III	IRCCS Ospedale San Raffaele – Milano

19	Hydroxychloroquine	PROTECT: A randomized study with Hydroxychloroquine versus observational support for prevention or early phase treatment of Coronavirus disease (COVID-19) - IRST 100.47	II	Ist. Scientifico Romagnolo per lo Studio e la Cura dei Tumori - IRST IRCCS - Meldola
20	Hydroxychloroquine Azithromycin	Open-label randomized controlled trial to evaluate efficacy and safety of the combination of hydroxychloroquine plus azithromycin versus hydroxychloroquine in patients with COVID-19 pneumonia (AZI-RCT-COVID19)	III	Università del Piemonte Orientale (UPO)
21	Hydroxychloroquine Tocilizumab Sarilumab Siltuximab Canakinumab, Baricitinib Methylprednisolone	Cumulative adaptive, multiarm, multistage and multicentre randomized clinical trial with immunotherapy for Moderate COVID-19 (the AMMURAVID trial)	Multifase	Società Italiana di Malattie Infettive e Tropicali (SIMIT)
22	Ivermectin	Randomized, Double-blind, Multi Centre Phase II, Proof of Concept, Dose Finding Clinical Trial on Ivermectin for the early Treatment of COVID-19	II	IRCCS Sacro Cuore Don Calabria, Verona
23	Interferon-β-1a	Randomized, controlled, open label, phase 2 clinical trial of Interferon-β-1a (IFNβ-1a) in COVID-19 patients	II	IRCCS Ospedale San Raffaele, Milano
24	Low-Molecular Weight Heparin	Randomised controlled trial comparing efficacy and safety of high versus low Low-Molecular Weight Heparin dosages in hospitalised patients with severe COVID-19 pneumonia and coagulopathy not requiring invasive mechanical ventilation (COVID-19 HD)	III	Azienda Ospedaliero-Universitaria di Modena
25	Mavrilimumab	A randomized, double blind, placebo-COntrolled trial of MavrilimumaB for Acute respiratory failure due To COVID-19 pneumonia with hyper-inflammation: the COMBAT-19 trial	II	IRCCS Ospedale San Raffaele – Milano
26	Pamrevlumab	An open-label, randomized, parallel-arm study investigating the efficacy and safety of intravenous administration of pamrevlumab versus standard of care in patients with COVID-19	II/III	UCSC - Roma
27	Polyvalent immunoglobulin	High dose intravenous polyvalent immunoglobulin (IVIG) in patients with early inflammatory COVID-19.	/	AUO Policlinico Umberto I Roma

28	Remdesivir	A Phase 3 Randomized Study to Evaluate the Safety and Antiviral Activity of Remdesivir (GS-5734™) in Participants with Moderate COVID-19 Compared to Standard of Care Treatment. (GS-US-540-5774 Study)	III	Gilead Sciences, Inc
29	Remdesivir	A Phase 3 Randomized Study to Evaluate the Safety and Antiviral Activity of Remdesivir (GS-5734™) in Participants with Severe COVID-19. (GS-US-540-5773 Study)	III	Gilead Sciences, Inc
30	Remdesivir<br>Hydroxychloroquine/<br>chloroquine<br>Lopinavir/Ritonavir<br>Interferone β1a	Studio controllato e randomizzato internazionale su trattamenti aggiuntivi per COVID-19 in pazienti ospedalizzati che ricevono tutti lo standard di cura locale	II<br>Disegno<br>adattativo	Organizzazione Mondiale della Sanità
31	Reparixin	Adaptive phase 2/3, randomized, controlled multicenter study on the efficacy and safety of Reparixin in the treatment of hospitalized patients with COVID-19 pneumonia (REPAVID-19)	II/III	Dompé farmaceutici Spa - Ospedale San Raffaele
32	Sarilumab	An adaptive phase 2/3, randomized, double-blind, placebo-controlled study assessing efficacy and safety of sarilumab for hospitalized patients with COVID-19 (Sarilumab COVID-19).	II/III	Sanofi-Aventis Recherche & Développement
33	Sarilumab	Randomized, open-label, multicenter phase 3 clinical trial aimed at comparing the clinical efficacy and safety of intravenous sarilumab in addition to the standard of care compared to the standard of care, in the treatment of patients with severe pneumonia due to COVID-19 (ESCAPE)	III	INMI "L. Spallanzani" - Roma
34	Sarilumab	Pilot study on the use of sarilumab in patients with covid-19 infection (COVID-SARI)	Studio pilota	ASST Fatebenefratelli Sacco
35	Selinexor	A Phase 2 Randomized Single-Blind Study to Evaluate the Activity and Safety of Low Dose Oral Selinexor (KPT-330) in Patients with Severe COVID-19 Infection (XPORT-CoV-1001)	II	Karyopharm Therapeutics Inc
36	Steroids<br>Heparin	Steroids and unfractionated heparin in critically ill patients with pneumonia from COVID-19 infection. A multicenter, interventional, randomized, three arms study design.	III	Azienda Ospedaliero-Universitaria di Modena

37	Tocilizumab	An open-label randomized multicenter study to evaluate the efficacy of early administration of Tocilizumab (TCZ) in patients with COVID-19 pneumonia	II	Azienda Unità Sanitaria Locale-IRCCS di Reggio Emilia
38	Tocilizumab	Multicenter study on the efficacy and tolerability of tocilizumab in the treatment of patients with COVID-19 pneumonia (TOCIVID-19)	II+ coorte osservazionale	Istituto Nazionale Tumori, IRCCS, Fondazione G. Pascale –Napoli
39	Tocilizumab	A randomized, double-blind, placebo controlled, multicenter study to evaluate the safety and efficacy of tocilizumab in patients with severe covid-19 pneumonia (Tocilizumab 2020-001154-22)	III	F. Hoffmann-La Roche Ltd
40	Tofacitinib Hydroxychloroquine	TOFAcitinib plus Hydroxycloroquine vs Hydroxycloroquine in patients with early onset SARS-CoV2 (COVID-19) interstitial pneumonia: a multicenter randomized controlled open label trial	II	Ospedali Riuniti di Ancona
41	Vaccine (GRAd-COV2)	A Phase 1, Dose-Escalation Study to assess the Safety and Immunogenicity of a COVID-19 Vaccine GRAd-COV2 in Healthy Adults and Elderly Subjects	I	ReiThera Srl

Appendix 3: clinical trials approved in Italy for COVID-19 (https://www.aifa.gov.it/en/web/guest/home). *SOC = standard of care*

**Randomized n (%)**	**Open-label n (%)**	**Controlled n (%)**			
34 (82,9)	30 (73,2)	34 (82,9)			
		**SOC n (%)**	**Placebo n (%)**	**Active comparator n (%)**	**No treatment n (%)**
		14 (41,2)	9 (26,5)	9 (26,5)	2 (5,9)

Appendix 4: Use of drugs outside approved indication in European countries.

ITALY

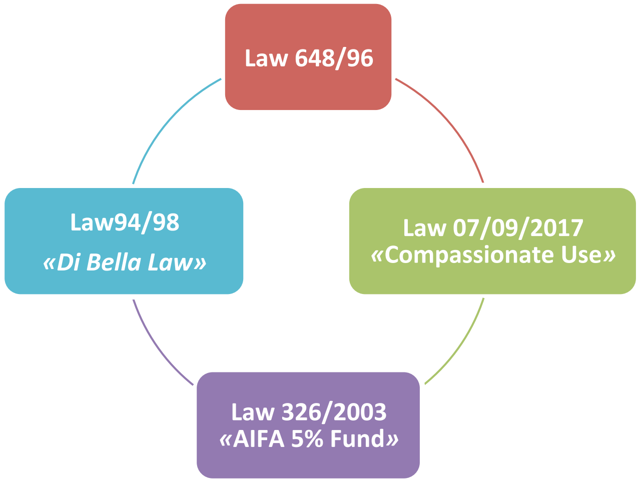

Obligations/requirements	Law 94/98	Law 648/96	Law 07/09/2017	Law 326/2003
**Lack of therapeutic options**	✓	✓ (Updated with the Law 79/2014: not mandatory in case of drugs at lower cost compared to on-label alternatives)	✓	✓
**Mandatory informed consent**	✓	✓	✓	✓
**Source of evidence**	At least phase II clinical trials (updated with the Financial Law 2008)	At least phase II clinical trials	At least phase II clinical trials (phase I for rare diseases)	Not specified
**Assumption of responsibility**	✓	/	✓	✓
**Periodic data transmission**	/	✓ to AIFA, Regions	✓ to AIFA	✓ to AIFA
**Cost of treatment**	Patient or hospital budget	NHS§	Company	AIFA 5% Fund*
**Formal authorization**	Case by case by Hospital health director (in some Regions)	Inclusion in the lists by AIFA	Case by case opinion by local Ethic Committe	Case by case opinion by AIFA

* *Established for the treatment of rare or severe diseases and supported by pharmaceutical companies*

§ National Health System

FRANCE

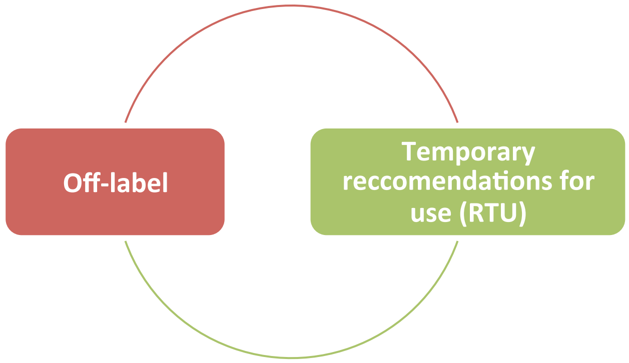

Obligations/requirements	Off-label	RTU
**Lack of therapeutic options**	✓	✓
**Mandatory informed consent**	✓	/
**Source of evidence**	Not specified (benefit/risk profile presumed to be favorable)	Not specified (benefit/risk profile presumed to be favorable)
**Assumption of responsibility**	/	/
**Periodic data transmission**	/	✓ to ANMS* (obligation for the MAH[§])
**Cost of treatment**	Not reimbursable	National Health Insurance
**Formal authorization**	Not necessary: prescribers are free to prescribe a medicine off-label	ANMS opinion

*****_Agence nationale de sécurité du médicament_

§ *Marketing Authorisation Holder*

GERMANY

Off-label

Obligations/requirements	Off-label
**Lack of therapeutic options**	✓
**Mandatory informed consent**	✓
**Source of evidence**	Not specified (evidence from scientific literature or authorisation of the indication in other countries)
**Assumption of responsibility**	/
**Periodic data transmission**	/
**Cost of treatment**	Health Insurance in case or severe disease
**Formal authorization***	Expert commission, GBA[§], Health Insurance company

**With respect to reimbursement*

[§] *Federal Joint Committee (Gemeinsamer Bundesausschuss)*

SPAIN

Off-label use regulated by the Royal Decree 1015/2009

Obligations/requirements	Off-label
**Lack of therapeutic options**	✓
**Mandatory informed consent**	✓
**Source of evidence**	Not specified
**Assumption of responsibility**	/
**Periodic data transmission**	✓<br>MAH should notify to the Agency any suspected adverse reactions and any information that may impact the recommendations
**Cost of treatment**	Regulated at regional and local level: case by case assessment and individual authorisation by medical director.
**Formal authorization**	Case by case not required; reccomendations for use/non use and monitoring by the National Agency
